# The polycomb group proteins, BMI-1 and EZH2, are tumour-associated antigens

**DOI:** 10.1038/sj.bjc.6603369

**Published:** 2006-10-03

**Authors:** J C Steele, E E Torr, K L Noakes, E Kalk, P A Moss, G M Reynolds, S G Hubscher, M van Lohuizen, D H Adams, L S Young

**Affiliations:** 1 Cancer Research UK Institute for Cancer Studies, University of Birmingham, Edgbaston, Birmingham B15 2TT, UK; 2 Department of Health, Wellington House, London SE1 8UG, UK; 3 Liver Research Laboratories, Institute for Biomedical Research, University of Birmingham Medical School, Birmingham B15 2TT, UK; 4 Department of Pathology, University of Birmingham Medical School, Edgbaston, Birmingham B15 2TT, UK; 5 Division of Molecular Genetics, The Netherlands Cancer Institute, Amsterdam 1066 CX, The Netherlands

**Keywords:** polycomb, tumour antigens, immunotherapy, cytotoxic T cell, regulatory T cell

## Abstract

We used SEREX technology to identify novel tumour-associated antigens in patients with primary hepatocellular carcinoma and found serological responses to the polycomb group (PcG) protein BMI-1, which is overexpressed in a range of different tumour types. Further studies identified T-cell responses to both BMI-1 and another PcG protein, EZH2, in cancer patients and at relatively lower levels in some normal donors. We next identified several CD8+ T-cell epitopes derived from *BMI-1* and *EZH2* and demonstrated that *EZH2*-derived peptides elicited more significant interferon-*γ* (IFN-*γ*) release than *BMI-1*-derived peptides. That CD8+ T cells were responsible for the observed responses was confirmed for *EZH2* by both IFN-*γ* capture assays and tetramer staining using an HLA-A0201-restricted, *EZH2*-derived YMSCSFLFNL (aa 666–674) epitope. The ability of YMSCSFLFNL (aa 666–674) to stimulate the *in vitro* expansion of specific T cells from peripheral blood lymphocytes was greatly enhanced when the CD25^+^ T-cell population was depleted. *EZH2*-specific cytotoxic T lymphocyte clones specific for two HLA-A0201 epitopes were generated and found to recognise endogenously processed *EZH2* in both HLA-matched fibroblasts and tumour cell lines. Given the widespread overexpression of PcG proteins in cancer and their critical role in oncogenesis, these data suggest that they may be useful targets for cancer immunotherapy.

Immunotherapeutic approaches to the treatment or prevention of cancer have flourished in recent years (Rosenberg, 2001; Rosenberg *et al*, 2004). This is largely due to the rapid identification of tumour-associated antigens (TAAs), which has been achieved by examining the specificity of existing T-cell responses (Stevanovic, 2002), or by using screening strategies such as serological identification of antigens by recombinant expression cloning (SEREX) (Pfreundschuh, 2000), proteomic analysis, gene expression profiling or combinations (Schultze and Vonderheide, 2001). The first tumour-specific vaccination was carried out in malignant melanoma which remains a valuable model for investigating cancer immunotherapy in humans (Saleh *et al*, 2005). With the growing number of TAAs identified, clinical studies have been expanded to other carcinomas (Stevanovic, 2002) and most of these studies used MHC class I-restricted, T-cell epitope peptides derived from TAAs to expand cytotoxic T cells (CTLs) for tumour destruction. Although this approach has the potential to induce antitumour immune responses, few studies have shown significant clinical responses (Rosenberg *et al*, 2004). Possible reasons for this are that the peptides chosen may not be naturally processed and presented by tumour cells, immune escape and unsolved questions about optimal clinical application. Recent studies implicate CD4^+^CD25^+^ regulatory T cells (T_REG_) within the tumour microenvironment in suppression of CD4+ and CD8+ T-cell activation and proliferation (Shevach *et al*, 2001). Increased numbers of both peripherally circulating and tumour-associated T_REG_ have been observed in tumour-bearing individuals with non-small lung, ovarian, breast, ovarian and hepatocellular carcinoma (HCC) (Woo *et al*, 2001, 2002; Liyanage *et al*, 2002; Ormandy *et al*, 2005; Unitt *et al*, 2005), and increased numbers of infiltrating tumour T_REG_ has been linked to a striking reduction in patient survival in ovarian cancer (Curiel *et al*, 2004).

With the exception of melanoma, the number of characterised TAAs for most tumours is limited and there is an urgent need to identify more TAAs and T-cell epitopes derived from their sequences. To be effective vaccines, T-cell epitopes must be naturally processed and presented antigenic targets capable of eliciting T-cell responses. The ultimate goal is to provide multiple tumour-associated epitopes presented by both MHC class I and class II molecules.

Here, we describe the identification by SEREX screening of a new TAA, the polycomb group (PcG) protein BMI-1, in HCC. BMI-1 and a related protein EZH2 are constituents of two distinct classes of PcG proteins. They are important transcriptional repressors that regulate gene activity by the formation of multiprotein complexes, called PcG bodies, and the organisation of chromatin into an inaccessible structure that cannot bind transcription factors (Shao *et al*, 1999). Interestingly, EZH2 appears to be one of the PcG proteins essential for BMI-1 recruitment to the PcG bodies (Hernandez-Munoz *et al*, 2005). PcG gene expression is tightly regulated and differs between different tissues and cell types. The proteins ensure correct embryonic development and are also major contributors to the regulation of haematopoiesis in the adult organism (Jacobs and van Lohuizen, 2002). *BMI-1* has been shown to be indispensable for the self-renewal of neural (Molofsky *et al*, 2003) and haematopoietic stem cells (Raaphorst, 2003), and it also has a key role in regulating the proliferative activity of normal stem and progenitor cells (Lessard and Sauvageau, 2003). *EZH2* is essential for cellular proliferation (Bracken *et al*, 2003). There is a clear connection between altered PcG gene expression and oncogenesis. Several mammalian PcG members have been implicated in the control of cellular proliferation and tumorigenesis (Jacobs and van Lohuizen, 2002), and altered expression of PcG genes is linked to transformation in cell lines and the induction of tumours in mutant mice (Satijn *et al*, 1997; Satijn and Otte, 1999). Several recent studies suggest that they also contribute to the development of cancer in humans. Aberrant PcG protein expression, such as overexpression, failure of downregulation or coexpression, has been reported in Hodgkin's lymphoma (Dukers *et al*, 2004), B-cell lymphoma (Raaphorst *et al*, 2004), mantle cell lymphoma (Bea *et al*, 2001; Visser *et al*, 2001), prostate cancer (Sellers and Loda, 2002; Varambally *et al*, 2002), breast carcinoma (Kleer *et al*, 2003; Raaphorst *et al*, 2003; Kim *et al*, 2004), HCC (Shi *et al*, 2005) and during lung carcinogenesis (Vonlanthen *et al*, 2001; Breuer *et al*, 2004). Some of these altered patterns of expression may be of diagnostic and prognostic relevance (Varambally *et al*, 2002; Raaphorst *et al*, 2003; Breuer *et al*, 2004; Kim *et al*, 2004). Our study shows that *BMI-1* and *EZH2* are overexpressed in several epithelial tumours and that both proteins are associated with both humoral and T-cell responses, suggesting that they represent a novel family of TAAs that might be potential targets for immunotherapy.

## MATERIALS AND METHODS

### Patients and subjects

Serum and/or peripheral blood mononuclear cells (PBMCs) were separated from heparinised blood samples (25–30 ml) taken from patients with a variety of epithelial tumours and from healthy donors. Histocompatibility locus antigen (HLA) class I tissue typing was carried out at the National Blood Service, Birmingham, UK. Tumour tissue was obtained from patients undergoing diagnostic biopsy or surgical resection. Normal tissue was taken from areas of tissue showing no tumour involvement. Approval was obtained from the South Birmingham Research Ethics Committee. Informed consent was obtained from all subjects.

### Synthetic peptides

CD8+ T-cell epitopes derived from both the BMI-1 and EZH2 proteins predicted to bind to HLA-A0201, HLA-B2702/05 and HLA-B440/3 were identified using the BioInformatics and Molecular Analysis Section website (Parker *et al*, 1994) (Table 1) and synthesised using fluoronylmethoxycarbonyl chemistry (Alta Bioscience, Birmingham, UK).

### Generation of BMI-1 and EZH2 constructs and recombinant adenoviruses

*BMI-1* or *EZH2* DNA sequences (a kind gift from Professor Thomas Jenuwein) were ligated into the pAdTrack-CMV vector containing the sequence for green fluorescent protein (GFP) and used directly in the nucleofection experiments. The empty vector encoding GFP was used as the negative control. Recombinant adenoviruses were produced by recombining the *EZH2*-containing pAdTrack-CMV vectors or the empty pAdTrack-CMV with the pAdEasy-1 vector and purified on caesium chloride gradients.

### SEREX screening of human sera against a HCC cDNA library

All serum samples were preabsorbed before SEREX screening. The primary screen was carried out using a commercially available HCC cDNA library (Novagen, Darmstadt, Germany). Serum from patients with HCC was tested initially and any positive clones screened against a panel of human serum from non-cancer donors. Samples were tested at a dilution of 1 : 1000 and positive plaques detected by alkaline phosphatase-conjugated goat anti-human IgG antibody (Sigma, Gillingham, Dorset, UK; 1 : 1000) and staining with 5-bromo-4-chloro-3-indolyl phosphate and nitroblue tetrazolium (BioRad, Richmond, USA). Positive plaques were re-screened to eliminate those encoding human immunoglobulin sequences. Phage DNA from positive clones was converted to stable plasmid subclones in BM25.8 cells and then transformed into JM109 cells before sequencing.

### Immunohistochemical staining for BMI-1

A variety of tumours, tumour cell lines and normal tissue were stained for the expression of BMI-1. Antigen retrieval was performed using the ALTER technique (Reynolds *et al*, 2002) and staining with the monoclonal antibodies to BMI-1 (229F6; Upstate, Watford, UK; 1 : 100) and 6C9 (1 : 10) was detected using Vector NovaRed (Vector Laboratories, Peterborough, UK) or DAB (Dako, Ely, Cambs, UK). The extent of staining was assessed semiquantitatively according to the proportion of cells with positively staining nuclei as follows: negative (<1% of cells positive), 1 (l–5%), 2 (5–10%), 3 (10–25%), 4 (25–50%) and 5 (>50%).

### Reverse transcription–PCR to detect EZH2 mRNA

RNA was isolated using the PolyATract system 1000 kit (Promega, Southampton, UK). First-strand cDNA was synthesised using SuperScript II reverse transcriptase with Oligo-dT priming (Invitrogen, Paisley, UK). Reverse transcription–PCR was performed for *EZH2* using forward and reverse primers 5′-CGGGGCTCCAAAAAGCATCTAT-3′ and 3′-GTCTGGATGGCTCTCTTGGCAA-5′, respectively, with a hot start of 94°C for 3 min followed by 30 cycles of 94°C, 30 s; 59°C, 30 s and 72°C, 45 s.

### Preparation of PBMCs and tumour-infiltrating lymphocytes

Peripheral blood mononuclear cells were separated from heparinised blood using Lymphoprep (Nycomed, Roskilde, Denmark) and cryopreserved. After thawing, cells were recovered overnight in RPMI 1640 medium containing 10% FCS (RPMI/10% FCS). CD4+ and CD8+ T-cell depletions were carried out using antibody-coated magnetic beads (Dynal, Paisley, UK) according to the manufacturer's instructions and depletion confirmed by flow cytometry. Tumour-infiltrating lymphocytes (TILs) were extracted from liver tumour tissue by homogenisation in RPMI/10% FCS followed by density gradient centrifugation through Lymphoprep.

### ELISPOT assay of interferon-*γ* release

Interferon-*γ* (IFN-*γ*) release was measured using a commercially available enzyme-linked immunospot (ELISPOT) kit (ELISPOT assay for human interferon-*γ*; Mabtech, Sweden) using cytokine capture and detection reagents according to the manufacturer's instructions. Peripheral blood mononuclear cells, CD4-depleted or CD8-depleted responder cells were seeded in triplicate wells and peptides added at 10 *μ*g ml^−1^. Negative control wells contained irrelevant peptide or diluted DMSO and positive control wells contained 10 *μ*g ml^−1^ PHA (Sigma) or an HLA-matched peptide epitope derived from Epstein–Barr virus (EBV). Spots were counted using an automated system.

### Interferon-*γ* release following nucleofection of PBMCs

Recovered PBMCs (2–10 × 10^6^) were nucleofected according to the manufacturer's instructions (Amaxa Biosystems, Cologne, Germany) with 5 *μ*g of plasmid DNA (pAdTrack-CMV vectors) encoding *BMI-1* and GFP, *EZH2* and GFP, or GFP alone as a negative control. Transfection efficiency was assessed after 24 h by fluorescent microscopy, and nucleofected cells were serially diluted into triplicate wells in 96-well U-bottomed plates (Nunc-Invitrogen, Paisley, UK) and left overnight at 37°C. Interferon-*γ* secretion in each well was determined by ELISA using 50 *μ*l of supernatant, capture and biotinylated detection monoclonal antibodies against IFN-*γ* (Endogen; 2G1 at 0.75 *μ*g ml^−1^ and B133.5 at 0.375 *μ*g ml^−1^, respectively), and then ExtrAvidin-Peroxidase (Sigma; 1 : 1000) followed by the addition of 3,3′,5,5′-tetramethylbenzidine substrate solution (Sigma). Interferon-*γ* standard solutions (31.25–2000 pg ml^−1^) were also run in triplicate.

### Detection and enrichment of EZH2-specific CD8+ T cells based on IFN-*γ* secretion

Detection and enrichment of *EZH2*-specific T cells was carried out using the Miltenyi Cytokine Secretion Assay PE kit for IFN-*γ* (Miltenyi Biotec, Surrey, UK) according to the manufacturer's instructions. Peripheral blood mononuclear cells isolated from HLA-A0201 donors were stimulated overnight at 37°C with the HLA-A0201-restricted, *EZH2* peptide YMCSFLFNL (aa 666–674) at 10 *μ*g ml^−1^. Negative control cultures contained diluted DMSO and positive controls staphylococcal enterotoxin B (Sigma) at 1 *μ*g ml^−1^. Interferon-*γ*-secreting cells were selected on automated columns. Pre- and postselected samples were analysed by flow cytometry using anti-human CD8-PC5 (Beckman Coulter, High Wycombe, Bucks, UK) with propidium iodide to gate out dead cells.

### Detection of EZH2-specific T cells using fluorogenic tetramers

Tetramers incorporating the HLA-A0201-restricted *EZH2* epitope YMCSFLFNL (aa 666–674) (Table 1) were produced as previously described (Altman *et al*, 1996). Peripheral blood mononuclear cells from HLA-A0201 donors were incubated with the tetramer and the FITC-conjugated CD8 antibody (T8; Beckman Coulter) for 20 min at 37°C and analysed by flow cytometry. Tetramer staining was performed on Peripheral blood mononuclear cells either directly *ex vivo*, or following 14 days in culture with the YMCSFLFNL (aa 666–674) peptide and cytokines human rIL-2 (Chiron, Oxford, UK; 10 U ml^−1^) and rIL-7 (Peprotech, London, UK; 20 ng ml^−1^) replenished every 3 days. In some experiments, CD25+ cells were depleted using the Magnetic Cell Sorting System (Miltenyi Biotech) before peptide stimulation *in vitro*. Depletions were verified by flow cytometry using anti-human CD25-PC5 (Beckman Coulter) and anti-human CD4-PE (Pharmingen, Cowley, Oxford, UK).

### Isolation of EZH2-specific cytotoxic T-cell clones

Peripheral blood mononuclear cells were stimulated with peptide (1–100 *μ*M) for 1–2 h at 37°C, washed, and resuspended in T-cell medium (RPMI 1640 containing 10% FCS, 2 mM glutamine and penicillin/streptomycin), supplemented with 25 ng ml^−1^ rIL-7 (Peprotech) and 10 U ml^−1^ rIL-2 after 3 days. Cultures were restimulated after 1 week using irradiated peptide-loaded autologous PBMCs (4000 Rads), and cloned by limiting dilution to 0.3, 3 and 30 cells well^−1^ on day 14. Each well also contained 1 × 10^4^ irradiated peptide-loaded autologous lymphoblastoid cells (LCL) generated by *in vitro* transformation of B cells using the EBV isolate B95.8 (Miller and Lipman, 1973), and 1 × 10^5^ irradiated PHA-stimulated mixed allogeneic buffy coat feeder cells (NBS, Birmingham). Cloning medium comprised T-cell medium containing 1% human AB serum (Gibco-Invitrogen, Paisley, UK), 100 U ml^−1^ rIL-2 and 30% supernatant from the MLA 144 cell line. Positive clones (initially screened against peptide-loaded autologous LCL target cells in an ELISA of IFN-*γ* release) were transferred to separate 2 ml wells in 1 ml of T-cell medium with 1 × 10^5^ irradiated peptide-loaded autologous LCL and 1 × 10^6^ irradiated PHA-stimulated mixed allogeneic buffy coat feeder cells. Wells were maintained by feeding twice weekly and were restimulated once a month.

Cytotoxicity was measured using chromium release assays as previously described (Lee *et al*, 1997). Target cells labelled with [^51^Cr]O_4_ were used at 2.5 × 10^3^ cells well^−1^ with T cells added at known effector : target ratios. Histocompatibility locus antigen-A0201-positive primary human fibroblasts (infected with recombinant adenoviruses RAd*EZH2* and RAdControl at a multiplicity of infection of 30 for 48 h before labelling) and tumour cell lines (LNCaP, HepG2 and MCF7) were employed as target cells in these assays. In some experiments, targets were cultured with IFN-*γ* (1000 U ml^−1^) for 48 h before assay and/or incubated with the anti-human HLA-A0201 monoclonal antibody BB7.2 (1 *μ*g well^−1^) for 1 h at 37°C after labelling and before the addition of T cells.

## RESULTS

### Identification of the PcG protein BMI-1 as a TAA in HCC using SEREX technology

Screening of an HCC cDNA library with serum from patients with HCC (not associated with viral hepatitis) using SEREX technology revealed an antigen which when sequenced was shown to be a member of the PcG family called *BMI-1*. Serum from nine HCC patients studied reacted strongly with *BMI-1* (Table 2; Supplementary Material), suggesting that immune responses to this antigen are frequent in HCC. The SEREX screening identified three sequences representing different cDNA clones of *BMI-1*, and all nine HCC serum samples screened had antibody responses to at least one of these. The majority of the noncancer serum samples also showed reactivity to *BMI-1*, but this was generally weaker as determined by intensity of plaque staining.

### Expression of BMI-1 and EZH2 in normal and tumour tissue

**Figure 1:**
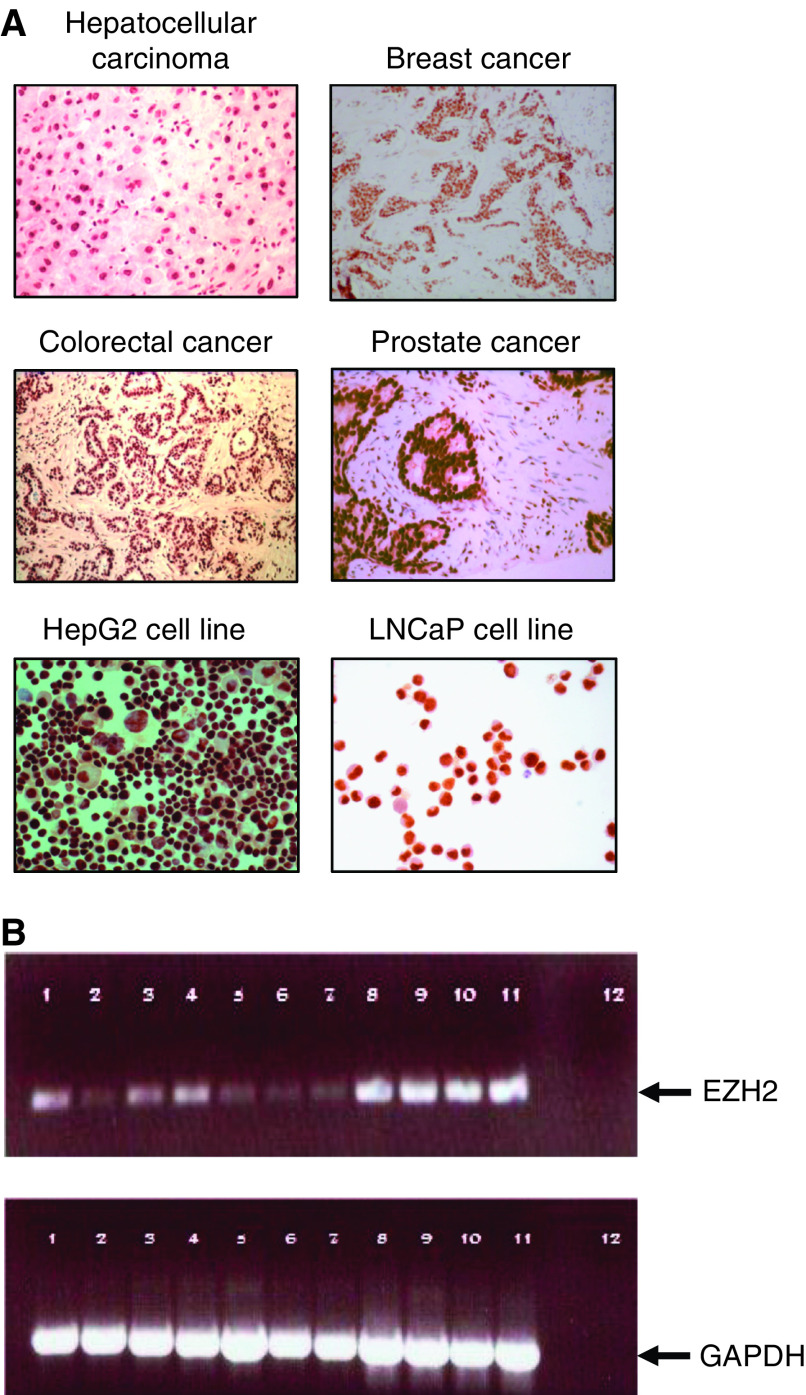
Expression of *BMI-1* and *EZH2*. (**A**) Immunohistochemical staining for BMI-1 expression on biopsy sections taken from different carcinomas and tumour cell line cytospins using the 229F6 monoclonal antibody. (**B**) Reverse transcription–PCR using *EZH2*-specific primers of various cell lines: 1, prostate cancer cell line (LNCaP); 2, normal human fibroblasts; 3, lymphoblastoid cell line(LCL)1; 4, LCL2; 5, LCL3; 6, LCL4; 7, cervical cancer cell line (Caski); 8, hepatoblastoma cell line (HepG2); 9, breast cancer cell line (MCF7); 10, breast cancer cell line (BT549); 11, breast cancer cell line (nDA-MB 436); 12, water.

Before embarking on a more detailed study of cellular immune responses to *BMI-1*, its expression was examined in a panel of tumour tissue samples and cell lines. All samples, both tissue sections and cell lines, displayed upregulation of the BMI-1 protein (Table 3; Supplementary Material). There was strong, specific, nuclear staining for BMI-1 involving at least 50% of cells in all of the cases studied and in some instances nearly 100% of the tumour cell population were positive (Figure 1A). There was minimal immunohistochemical staining for BMI-1 (<1% of cells) in normal tissues from breast, stomach, colon or skin (Table 2; Supplementary Material). Three of the four normal liver samples showed slightly more extensive nuclear staining involving <10% of hepatocytes. Occasional infiltrating lymphocytes were also positive in normal tissue specimens. There is currently no commercially available antibody for *EZH2*, so we used RT–PCR to analyse *EZH2* mRNA expression in tumour cell lines. High-level expression of *EZH2* mRNA was observed in the prostate cancer cell line LNCaP, the HepG2 hepatoblastoma cell line and three breast cancer cell lines MCF7, BT549 and nDA-MB436. This confirms previous studies demonstrating upregulation of *EZH2* expression in tumours of patients with breast cancer (Kleer *et al*, 2003; Raaphorst *et al*, 2003; Kim *et al*, 2004). Variable expression was seen in a number of EBV-transformed LCL, with low expression of *EZH2* mRNA in the cervical carcinoma cell line Caski, and in normal fibroblasts (Figure 1B).

### Systemic T-cell responses to BMI-1 and EZH2 can be detected in patients with solid tumours

We tested for immune responses to *BMI-1* and *EZH2* by measuring IFN-*γ* production from PBMC cultures nucleofected with plasmid DNA (pAdTrack-CMV) encoding the EZH2 and BMI-1 proteins. Background levels of IFN-*γ* production following nucleofection were assessed using the empty pAdTrack-CMV vector, which encodes GFP alone, and were generally low. The presence of GFP in the pAdTrack-CMV vectors expressing *BMI-1* and *EZH2* also allowed an assessment of the nucleofection efficiency, which was generally between 1 and 2%.

**Figure 2:**
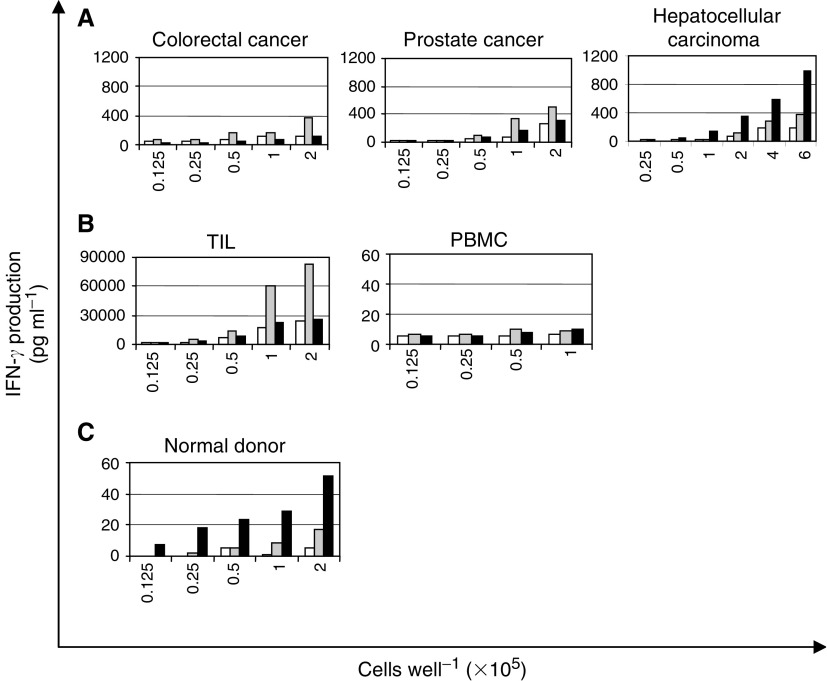
Interferon-*γ* production by PBMC and TIL cultures from cancer patients and normal donors following nucleofection with plasmid DNA expressing BMI-1 and EZH2 proteins. Peripheral blood mononuclear cells samples from patients with a variety of (**A**) epithelial tumours and (**C**) normal donors were nucleofected with 5 *μ*g of plasmid DNA (pAdTrack-CMV) encoding either *BMI-1* and GFP (░), or *EZH2* and GFP (▪) or GFP alone (□). (**B**) Results from paired cultures of TILs and PBMCs from a patient with hepatocellular carcinoma are also shown. Interferon-*γ* production was measured in the supernatants of serially diluted PBMC cultures using an ELISA. The results shown are means of triplicate wells and standard errors were always less than 10%.

Responses to *BMI-1* were seen in one out of 10 HCC patients, two out of six prostate cancer patients and three out of five colorectal cancer patients. *EZH2* reactivity was observed in three out of ten HCC patients, one out of five colorectal cancer patients and in none of the prostate cancer samples. One sample from both the HCC group and the colorectal cancer group demonstrated T-cell reactivity to both antigens. No responses were seen to either antigen in the three breast cancer patients investigated. Although the numbers investigated in each group were small, there appeared to be a dominant response to *BMI-1* in the patients with colorectal and prostate cancer, whereas stronger reactivity to *EZH2* was more common among the HCC patients. Typical responses from each group are shown in Figure 2A where it can be seen that levels of IFN-*γ* production were reasonably high. Figure 2B shows the results of a paired PBMCs and TILs population from a patient with HCC. No T-cell reactivity was seen in the nucleofected PBMC cultures to either *BMI-1* or *EZH2*, but interestingly IFN-*γ* production was extremely high in the TILs culture expressing *BMI-1*, suggesting that immune responses may be compartmentalized in the tumour. These data demonstrate that T-cell responses to both *BMI-1* and *EZH2* are evident in cancer patients.

**Figure 3:**
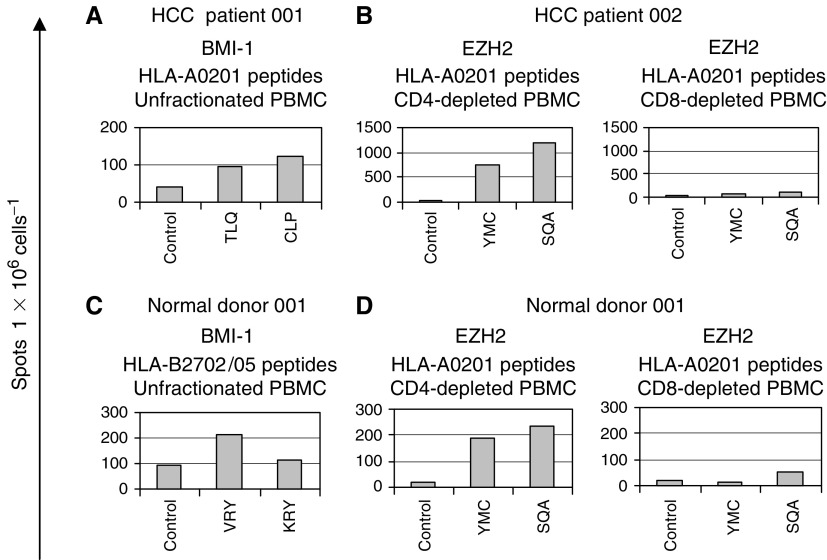
ELISPOT responses in patients with hepatocellular carcinoma (HCC) and normal donors to predicted CD8+ T-cell epitope peptides. Unfractionated PBMCs or CD4- and CD8-depleted responder cell populations were screened against 9-mer peptides derived from BMI-1 and EZH2 proteins predicted to bind to the HLA class I molecules HLA-A0201, HLA-B2702/05 and HLA-B4403 (Table 1) using an ELISPOT of interferon-*γ* release. The figure shows positive responses to the HLA-A0201-restricted *BMI-1* peptides TLQDIVYKL (aa 74–82; TLQ) and CLPSPSTPV (aa 271–279; CLP) in HCC patient 001 (**A**), to the HLA-B2702/05-restricted *BMI-1* peptides VRYLETSKY (aa 44–52; VRY) and KRYLRCPAA (aa 161–169; KRY) in normal donor 001 (**C**), and to the HLA-A0201-binding *EZH2* peptides YMCSFLFNL (aa 666–674; YMC) and SQADALKYV (aa 734–742; SQA) in HCC patient 002 (**B**) and normal donor 001 (**D**). Peptides were added to a final concentration of 10 *μ*g ml^−1^ and results obtained from background control wells containing cells and diluted DMSO are shown. Results are the mean of triplicate wells, and are expressed as the number of spots obtained per 1 × 10^6^ cells added to each well. Standard errors were less than 10%. For depleted populations, these figures were calculated following FACS analysis after depletion.

To confirm the T-cell responses in patients with HCC, we used CD8+ T-cell peptide epitopes derived from both the BMI-1 and EZH2 proteins predicted to bind to HLA-A0201, HLA-B2702/05 and HLA-B4402/03 molecules (Table 1) in ELISPOT assays of IFN-*γ* release. Eleven patients of the appropriate HLA type were investigated and typical results are shown in Figure 3A and B. Responses to *BMI-1*, all directed against HLA-A201-binding peptides, were detected in three individuals with one showing reactivity to the CLPSPSTPV (aa 271–279), one to TLQDIVYKL (aa 74–82) and one to both peptides (Patient 001 shown in Figure 3A). Two out of the 11 gave a positive response to the HLA-A201-binding epitopes YMCSFLFNL (aa 666–674) and SQADALKYV (aa 734–742) derived from *EZH2*. To confirm that these were CD8+ T-cell responses, we compared unfractionated PBMCs with CD4- or CD8-depleted responder cell populations (FACS analysis confirmed depletion of greater than 99% of the appropriate T-cell subset) (Patient 002 shown in Figure 3B). The magnitude of the responses to the *EZH2* peptides was generally greater than those to *BMI-1*.

### Weaker T-cell responses to BMI-1 and EZH2 are also seen in normal donors

We also investigated T-cell reactivity to *BMI-1* and *EZH2* in normal laboratory donors by measuring IFN-*γ* production from PBMC cultures expressing BMI-1 and EZH2 proteins following nucleofection (as above). Background levels of IFN-*γ* production to the control GFP-expressing plasmid were again low and nucleofection efficiency was consistently 1–2%. Two out of the eight normal samples investigated demonstrated reactivity to *BMI-1*, and four to *EZH2*. Interferon-*γ* production was generally 10–20 times lower than with samples from patients with cancer (Figure 2C).

In the ELISPOT assay, two out of 10 normal donors showed a response to *BMI-1*-predicted peptides. One individual demonstrated reactivity to the HLA-A201-binding peptide TLQDIVYKL (aa 74–82), and the other to the HLA-B2702/05-binding peptides VRYLETSKY (aa 44–52) and KRYLRCPAA (aa 161–169; Donor 001 shown in Figure 3C). *EZH2* reactivity was observed in two out of seven donors. Both showed a positive response to the HLA-A201-binding epitopes YMCSFLFNL (aa 666–674) and SQADALKYV (aa 734–742), which depletion experiments confirmed to be CD8+ T-cell reactivity (Donor 001 shown in Figure 3D), and one of these individuals also showed weaker reactivity against two putative HLA-B4403-restricted peptides EELFVDYRY (aa 725–733) and KESRPPRKF (aa 210–218). The magnitude of the responses obtained from normal donors for both *BMI-1* and *EZH2* was less than that for HCC patients.

**Figure 4:**
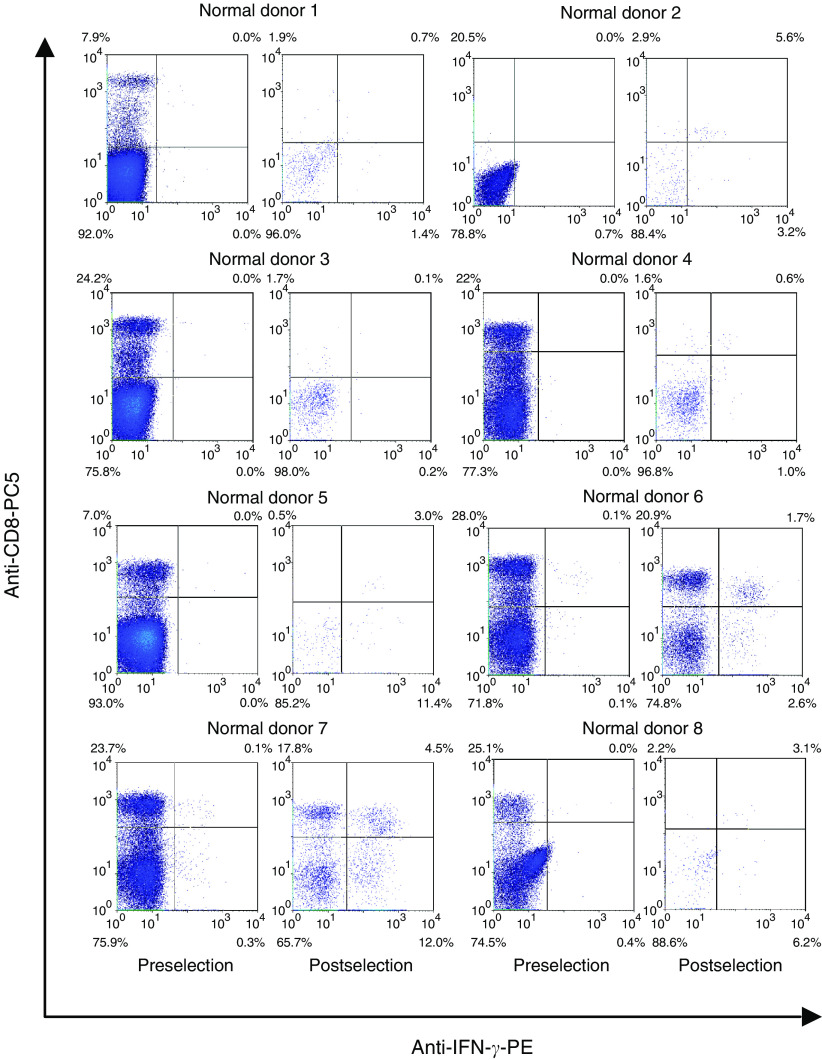
Detection and enrichment of IFN-*γ* secreting cells from normal donors. Peripheral blood mononuclear cells obtained from normal HLA-A0201-positive laboratory donors were stimulated *in vitro* overnight with the HLA-A0201-restricted *EZH2* peptide YMCSFLFNL (aa 666–674) at 10 *μ*g ml^−1^. Interferon-*γ*-secreting cells were detected using a PE-labelled IFN-*γ* detection antibody, enriched on anti-PE magnetic microbeads and selected on automated columns. The figure shows flow cytometric analysis of pre- and postselected samples (stained using an anti-human CD8-PC5 antibody) from the same donor. Dead cells have been gated out after staining with propidium iodide.

**Figure 5:**
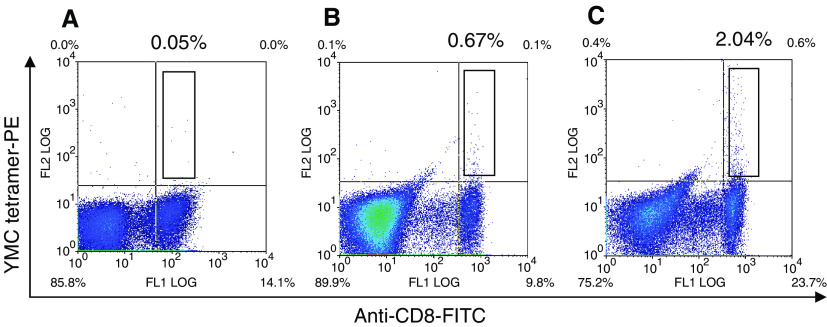
Staining of normal PBMCs with theYMCSFLFNL (aa 666–674) tetramer. Peripheral blood mononuclear cells isolated from an HLA-A0201-positive normal laboratory donor were stained with a tetramer incorporating the HLA-A0201-restricted *EZH2* epitope YMCSFLFNL (aa 666–674) and an FITC-conjugated surface CD8 antibody directly *ex vivo* (**A**) and following a 14-day *in vitro* restimulation step with the YMCSFLFNL (aa 666–674) peptide (**B**). Peptide restimulation was also carried out on the same population of PBMCs after depletion of CD25^+^ T cells (**C**). The values shown are the percentage of CD8+ T cells staining positive with the tetramer.

A cytokine secretion assay based on IFN-*γ* was employed for the detection and enrichment of peripheral blood T cells from normal donors that responded to the *EZH2*-predicted epitope YMCSFLFNL (aa 666–674). Eight HLA-A0201-positive laboratory donors were tested and significant enrichment of *EZH2*-specific CD8^+^ T cells was seen in five of these (Figure 4). We then used MHC class I tetramers incorporating the HLA-A0201-restricted *EZH2* epitope YMCSFLFNL (aa 666–674) to further demonstrate systemic T-cell reactivity to EZH2. Peripheral blood mononuclear cells isolated from seven HLA-A0201-positive healthy laboratory donors were stained directly *ex vivo* with the tetramer, and between 0.05 and 0.75% (median 0.15%) CD8+ T cells stained. In four out of six of the normal PBMC samples, the percentage of tetramer-positive CD8+ T cells could be increased by carrying out a 14-day *in vitro* incubation with the YMCSFLFNL (aa 666–674) peptide (range 0.03–1.04%; median 0.585%) (Figure 5A and B).

### Depletion of CD25+ T cells allows greater *in vitro* expansion of EZH2-specific T cells

We hypothesized that *EZH2*-specific T cells might be suppressed *in vivo* by regulatory T cells. In order to test this, CD25+ T-cell depletion was carried out on six HLA-A0201-positive normal PBMC samples (99% of CD4+CD25+ T cells were depleted) and subsequent *in vitro* restimulation with the YMCSFLFNL (aa 666–674) peptide carried out for 14 days. In two out of six of the samples, a marked expansion of YMCSFLFNL (aa 666–674)-tetramer positive cells was detected. The data for one of these are shown in Figure 5, where it can be seen that 0.67% of cells within the CD8+ T-cell fraction were tetramer positive without CD25 depletion as opposed to 2.04% with CD25 depletion.

### EZH2-specific T-cell clones are cytotoxic against endogenously processed antigen and tumour cell lines

**Figure 6:**
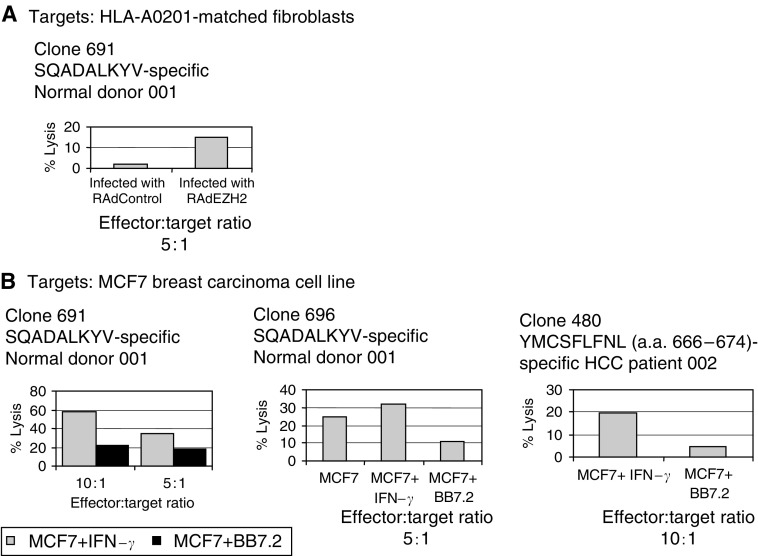
Cytotoxicity of *EZH2*-specific cytotoxic T-cell clones 691, 696 and 480 derived from normal donor 001 and HCC patient 002 against (**A**) HLA-A0201-matched primary human fibroblasts infected with recombinant adenoviruses (either RAd*EZH2* or RAdControl) and (**B**) the HLA-A0201-positive breast carcinoma cell line MCF7. Chromium release assays employed 2.5 × 10^3^ target cells well^−1^. Effector : target cell ratios and additions of either IFN-*γ* or monoclonal antibody BB7.2 are indicated on individual graphs. The results shown are means of triplicate wells and standard errors were less than 10%.

*EZH2*-specific CTL clones specific for the HLA-A0201-restricted peptide epitopes YMCSFLFNL (aa 666–674) and SQADALKYV (aa 734–742) were isolated from both HLA-A0201-positive HCC patients and normal donors by peptide restimulation *in vitro*. Antigen-specific cytotoxicity was observed against HLA-A0201-matched fibroblasts infected with a recombinant adenovirus encoding *EZH2* (RAd*EZH2*) (Figure 6A) and the breast carcinoma cell line MCF7, which naturally expresses *EZH2* (Figure 6B). Greater levels of lysis were observed following incubation of the tumour cells with IFN-*γ* and T-cell reactivity was either greatly reduced or effectively blocked following incubation of the target cells with the anti-HLA-A0201 monoclonal antibody BB7.2. Low levels of killing were observed against the HLA-A0201-positive LNCaP prostate cancer cell line and HepG2 hepatoblastoma cells (data not shown).

## DISCUSSION

We employed SEREX technology using a cDNA library to identify new TAAs associated with liver cancer. The SEREX approach was chosen because the method identifies antigens that are of immunological relevance, as their detection requires patients to have generated antibodies against them. SEREX has previously detected tumour antigens with epitopes for cellular immune responses, the best-known example being *NY-ESO-1* (Gnjatic *et al*, 2002). In our study, screening of an HCC cDNA library with serum from patients with nonvirally induced HCC revealed different antigens, with a particularly strong response from all patient samples to the PcG protein BMI-1 (Table 2; Supplementary Material). This strong reactivity, together with the fact that the PcG proteins are known to be vitally involved in transcriptional control and carcinogenesis in humans, and may therefore be less susceptible to the development of ‘immune escape’ variants, made *BMI-1* an attractive candidate for validation as a target for cancer immunotherapy. Recent studies showing that another polycomb protein, EZH2, which is essential for BMI-1 recruitment to the polycomb body in the nucleus (Hernandez-Munoz *et al*, 2005), is also highly expressed in many cancers and led us to extend our studies to include both *BMI-1* and *EZH2*. As expression of these two proteins is often mutually exclusive, a sensible approach may be an attempt to target both.

Consistent with recent studies demonstrating overexpression of PcG genes in cancer (Vonlanthen *et al*, 2001; Sellers and Loda, 2002; Kleer *et al*, 2003; Raaphorst *et al*, 2003; Breuer *et al*, 2004; Kim *et al*, 2004; Shi *et al*, 2005), we found that both *BMI-1* and *EZH2* are strongly expressed by a variety of tumours (Table 3; Supplementary Material; Figure 1). This broad upregulation in cancer makes them attractive immunotherapeutic targets for malignant disease. Low level expression of both proteins was also observed in some normal tissues, which is consistent with the reported role of *BMI-1* and *EZH2* in self-renewal and proliferative activity of stem cells and their progenitors (Bracken *et al*, 2003; Molofsky *et al*, 2003). The low-level restricted expression on normal tissues suggests that effective immune responses against *BMI-1* and *EZH2* may be compromised by self-tolerance and that stimulating immune reactivity to these proteins may be accompanied by autoimmune side effects. However, the majority of cancer-specific immunotherapies have employed self-antigens and autoimmunity has not proven to be a significant problem in most clinical studies (Gilboa, 2004).

**Figure 7:**
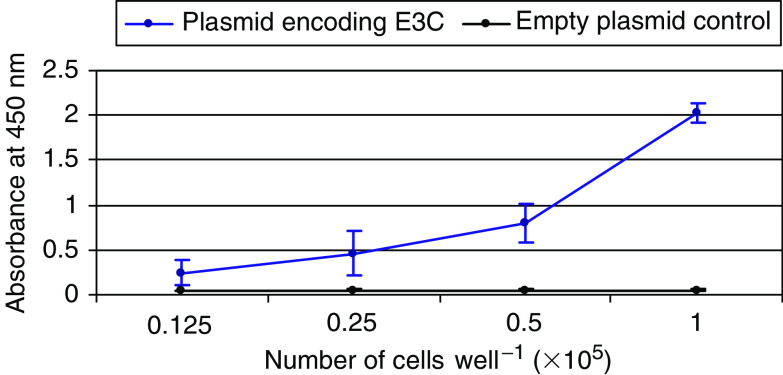
Interferon-*γ* production by a PBMC culture from a normal donor following nucleofection with plasmid DNA expressing the E3C protein from EBV protein. Peripheral blood mononuclear cells were nucleofected with 5 *μ*g of plasmid DNA encoding E3C or the empty vector and IFN-*γ* production was measured after 48 h incubation.

Because CTL responses are important for tumour destruction, the demonstration of CTL responses against PcG proteins would strengthen their potential as tumour antigens. Initially, we looked for IFN-*γ* secretion from PBMCs that had been nucleofected with plasmids encoding either the BMI-1 or EZH2 proteins. A system optimised for the transfection of dendritic cells was employed so that monocytes and macrophages would be preferentially nucleofected. Fluorescence-activated cell sorter (FACS) analysis revealed that T cells and B cells were also expressing the proteins and that transfection efficiencies were around 1–2%. Earlier experiments using EBV antigens had established that these conditions provided effective antigen processing and presentation and reliable antigen-specific IFN-*γ* responses using low numbers of cells (Figure 7). Patients with HCC, prostate cancer, colorectal cancer and breast cancer were studied, and with the exception of breast cancer, T-cell reactivity to *BMI-1* and/or *EZH2* was observed in all groups. Predicted CD8+ T-cell epitopes derived from *BMI-1* and *EZH2* also elicited T-cell responses as assessed by IFN-*γ* release confirming the presence of CD8 responses against these proteins in patients with cancer. These data also confirmed that these epitopes identified by predictive algorithms are likely to represent naturally presented epitopes and may therefore be significant for the design of peptide-based vaccines.

The SEREX screening demonstrated reactivity to cDNA clones representing *BMI-1* in serum obtained from most of the noncancer patients, and T-cell assays also detected *BMI-1*- and *EZH2*-specific T-cell responses in normal individuals using either nucleofection of PBMCs or ELISPOT assays with T-cell epitopes. Enrichment of IFN-*γ* secreting CD8+ T cells specific for YMCSFLFNL (aa 666–674) was possible in five out of eight normal donors (Figure 4B) and systemic T-cell responses to *EZH2* were demonstrated in HLA-A0201-positive normal donors by YMCSFLFNL (aa 666–674)-tetramer staining. Significantly, with the majority of these samples, the percentage of CD8+ T cells, which stained positive, could be increased by carrying out an *in vitro* restimulation step with the YMCSFLFNL (aa 666–674) peptide. The fact that these *EZH2*-specific T cells could be expanded *in vitro* is encouraging because it opens up the possibility of either adoptive T-cell therapy or expansion *in vivo* by vaccination.

It is perhaps not surprising that responses to *BMI-1* and *EZH2* were detected in normal donors, as there are likely to be autoreactive T cells of low avidity present in the mature T-cell population for the majority of self-antigens. This has been demonstrated for other TAAs (Marincola *et al*, 1996). In fact, it is the vulnerability of the immune system to activate anti-self immunity that is the basis for developing useful cancer-specific immunotherapies and it is of note that, although the frequency of detected responses to PcG proteins was similar between normal and cancer patients, the normal donors consistently showed a weaker immune response. It is intriguing to speculate that immune responses to PcG proteins could be involved in the control of normal haematopoeisis and/or regulating stem cell renewal.

The study revealed differences in responses between *BMI-1* and *EZH2* with both cancer patients and normal donors. Interferon-*γ* production following nucleofection was more readily detected in response to *BMI-1* in the patients with colorectal and prostate cancer, whereas the HCC patients tended to have stronger responses to *EZH2*. This dominant response to *EZH2* in the HCC group was also apparent with the ELISPOT assays, where the magnitude of the responses to the *EZH2* peptides was generally greater than those to *BMI-1*. *EZH2* T-cell reactivity was more common than *BMI-1* reactivity among normal donors also, suggesting that *BMI-1* may be less immunogenic than *EZH2*. *BMI-1* is more widely expressed on stem cell populations (Lessard and Sauvageau, 2003; Raaphorst, 2003) and so represents an important regulatory antigen associated with a more ‘primitive’ cell type. It could be that the immune system is, therefore, more tolerant to *BMI-1* than *EZH2*. The strong response to *BMI-1* among some of the patients with colorectal and prostate cancer may be due to the pattern of aberrant expression of the polycomb proteins by these tumours. Overexpression of *BMI-1* may result in a break in tolerance and reactivation of autoreactive T cells by exposure to high concentrations of antigen.

*EZH2*-specific T-cell clones were obtained by *in vitro* peptide restimulation using the HLA-A0201-restricted CD8+ T-cell epitope YMCSFLFNL (aa 666–674). These clones were able to recognize and kill target cells overexpressing *EZH2* following infection with a recombinant adenovirus, and also the HLA-A0201-matched breast tumour cell line MCF7. Blocking experiments with the BB7.2 monoclonal antibody against HLA-A0201 confirmed that this recognition and cytotoxicity was MHC class I-restricted. It was encouraging that T cells cloned in response to an *EZH2*-derived peptide could recognize endogenously processed and presented antigen, suggesting that *EZH2*-specific T cells would be able to recognize and kill tumour cells. Functional *EZH2*-specific T cells have previously been isolated from patients with prostate cancer using HLA-A2402-restricted peptides. These effectors were shown to be cytotoxic against the C1R-A24 subline of C1R lymphoma and also the prostate cancer cell line LNCaP, which had been stably transfected with the HLA-A2402 gene (Ogata *et al*, 2004).

It has been suggested that SEREX identifies self-antigens that elicit naturally occurring CD4+CD25+ regulatory T cells (T_REG_) which maintain and regulate immunological homeostasis (Nishikawa *et al*, 2005) and our study supports this hypothesis. In experiments using the *EZH2*-derived HLA-A0201-restricted peptide YMCSFLFNL (aa 666–674), we were able to show that depletion of CD25^+^ T cells promotes the expansion of YMCSFLFNL (aa 666–674)-tetramer positive cells in normal PBMC cultures undergoing peptide restimulation *in vitro*. This suggests that T_REG_ do play a role in suppressing at least CD8+ T-cell reactivity to *EZH2*. In tumours, where numbers of T_REG_ may be greatly increased, this immunosuppressive effect may be highly significant.

We have demonstrated that the PcG proteins BMI-1 and EZH2 represent promising target antigens for cancer immunotherapy. They are expressed on a wide variety of tumours where they appear essential to the oncogenic process and show restricted expression in normal tissue. They are naturally immunogenic and *EZH2*-specific T cells can be reactivated and expanded *in vitro* by a combination of antigen stimulation and T_REG_ depletion. Most importantly, these T cells can recognize endogenously processed antigen and are cytotoxic against tumour cell lines naturally expressing *EZH2*, suggesting that PcG proteins are a novel group of TAA with potential as antigens for immunotherapy in a wide range of cancers.

## Figures and Tables

**Table 1:** Peptide sequences from *BMI-1* and *EZH2* predicted to bind HLA class I molecules

**HLA class I restriction element**	***BMI-1* a**	**Amino-acid position**	***EZH2^b^***	**Amino-acid position**
A0201	TLQDIVYKL	74–82	YMCSFLFNL	666–674
	CLPSPSTPV	271–279	SQADALKYV	734–742
				
B2702/05	VRYLETSKY	44–52	KRFRRADEV	30–38
	KRYLRCPAA	161–169	YRYSQADAL	731–739
				
B4403	YEEEPLKDY	195–203	EELFVDYRY	725–733
	KEEVNDKRY	155–163	KESRPPRKF	210–218

Peptides were predicted using the BioInformatics and Molecular Analysis Section (BIMAS) website (Parker *et al*, 1994).

a *BMI-1* accession number NM_005180.

b *EZH2* accession number NM_004456.
