# Supplementary material for: The polycomb group proteins, BMI-1 and EZH2, are tumour-associated antigens
Source: Br J Cancer. 2006 Oct 3;95(9):1202–11. doi: 10.1038/sj.bjc.6603369 (PMC2360579; doi:10.1038/sj.bjc.6603369)
Supplement: Table 2; Supplementary Material [file 95-6603369x1.doc]

**Table 2.** Serex screening of human serum from HCC and non-cancer patients against an HCC cDNA library

| Clone No | Accession Number | Description | HCC serum reactivity | Reactivity | Non-cancer serum reactivity | Reactivity |
| --- | --- | --- | --- | --- | --- | --- |
| 5 | NM_152437 | Hypothetical protein DKFZp761B128 | 7/9 | + | 4/10 | + |
| 9 | NM_181659 | Nuclear receptor coactivator 3 (NCOA3) | 2/9 | + | 1/10 | + |
| 10 | NM_001009820 | Small nuclear ribonucleoprotein 70KDa polypeptide (SNRP70) | 4/9 | + | 3/10 | + |
| 11 | NM_012067 | Aldo-keto reductase family 7, member A3 (Aflatoxin aldehyde reductase) (AKR7A3) | 2/9 | ++ | 3/10 | + |
| 12 | NM_000918 | Procollagen-proline, 2-oxoglutarate 4-dioxygenase (proline 4-hydroxylase) beta polypeptide (protein disulfide isomerase thyroid hormone binding protein P55) | 1/9 | + | 4/10 | + |
| 18 | NM_002356 | Myristoylated alanine-rich protein kinase C substrate (MARCKS) | 4/9 | ++ | 7/10 | + |
| 45 | NM_005180 | B Lymphoma moloney murine leukaemia virus insertion region (BMI-1) | 9/9 | +++ | 9/10 | ++ |
| 51 | X17625 | rRNA primary transcript 5' external transcribed spacer | 5/9 | + | 1/10 | + |
| 53 | NM_012235 | SREBP cleavage-activating protein | 2/9 | ++ | 4/10 | + |
| 54 | NM_001402 | Eukaryotic translation elongation factor 1, alpha 1 (EEF1A1) | 2/9 | ++ | 4/10 | + |
| 56 | NM_005180 | B Lymphoma moloney murine leukaemia virus insertion region (BMI-1) | 8/9 | ++ | 8/10 | ++ |
| 71 | NM_003088 | Fascin homolog 1, actin-bundling protein (FSCN1) | 2/9 | ++ | 4/10 | ++ |
| 108 | NM_005180 | B Lymphoma moloney murine leukaemia virus insertion region (BMI-1) | 8/9 | +++ | 9/10 | ++ |
| 109 | NM_002884 | Member of the RAS oncogene family (RAP1A) | 2/9 | ++ | 6/10 | + |
| 110 | NM_002945 | Replication protein A1 (RPA1) | 5/9 | + | 5/10 | + |
| 111 | NM_002884 | Member of the RAS oncogene family (RAP1A) | 4/9 | + | 3/10 | ++ |
| 118 | NM_001832 | Pancreatic colipase (CLPS) | 1/3 | + | 3/10 | + |
| 119 | AL691432 | DNA sequence from clone RP11-345P4 | 2/3 | ++ | 2/10 | ++ |

Shaded rows indicate the three different cDNA clones of BMI-1 strongly recognised by serum from HCC patients.

The strength of the serum reponses to positive clones has been graded (+, ++ or +++) according to the intensity of plaque staining after development with the alkaline phosphatase substrate.
