# Supplementary material for: The polycomb group proteins, BMI-1 and EZH2, are tumour-associated antigens
Source: Br J Cancer. 2006 Oct 3;95(9):1202–11. doi: 10.1038/sj.bjc.6603369 (PMC2360579; doi:10.1038/sj.bjc.6603369)
Supplement: Table 3; Supplementary Material [file 95-6603369x2.doc]

| **Tissue** | **No. of samples analysed** | **Number of samples scoring** | | | | | | **Tissue** | **No. of samples analysed** | | **Number of samples scoring** | | | | | | | | |
| --- | --- | --- | --- | --- | --- | --- | --- | --- | --- | --- | --- | --- | --- | --- | --- | --- | --- | --- | --- |
|  |  | **0** | **1** | **2** | **3** | **4** | **5** |  |  |  | **0** | | **1** | | **2** | | **3** | **4** | **5** |
| **TUMOUR** | | | | | | | | **NORMAL** | | | | | | | | | | | |
| HCC | 6 | 0 | 0 | 0 | 0 | 2 | 4 | Liver | | 4 | | 1 | | 2 | | 1 | 0 | 0 | 0 |
| HCC metastases | 4 | 0 | 0 | 0 | 0 | 0 | 4 | Breast | | 2 | | 2 | | 0 | | 0 | 0 | 0 | 0 |
| PTLD | 5 | 0 | 0 | 0 | 0 | 0 | 5 | Stomach | | 2 | | 2 | | 0 | | 0 | 0 | 0 | 0 |
| Prostate | 5 | 0 | 0 | 0 | 0 | 0 | 5 | Colon | | 2 | | 2 | | 0 | | 0 | 0 | 0 | 0 |
| Breast | 5 | 0 | 0 | 0 | 0 | 0 | 5 | Normal skin | | 2 | | 2 | | 0 | | 0 | 0 | 0 | 0 |
| Stomach | 5 | 0 | 0 | 0 | 0 | 0 | 5 | **CELL LINES** | | | | | | | | | | | |
| Colon | 5 | 0 | 0 | 0 | 0 | 0 | 5 | Hep G2 | | 1 | | 0 | | 0 | | 0 | 0 | 0 | 1 |
| Lung | 4 | 0 | 0 | 0 | 0 | 0 | 4 | LNCaP | | 1 | | 0 | | 0 | | 0 | 0 | 0 | 1 |
| Skin | 5 | 0 | 0 | 0 | 0 | 0 | 5 | LCL | | 5 | | 0 | | 0 | | 0 | 0 | 0 | 5 |
| Cervical | 3 | 0 | 0 | 0 | 0 | 0 | 3 |  | |  | |  | |  | |  |  |  |  |

**Table 3.** BMI-1 expression in different carcinomas, tumour cell lines and normal tissue

The table indicates the number of each type of sample analysed, and the number of samples within these groups which scored a particular grade of staining. Samples were graded according to the percentage of cells positive. In tumour samples only malignant cells were counted. Positive staining was scored as follows: 0 (<1%); 1 (l – 5 %); 2 (5 –1 0%); 3 (10 – 25%); 4 (25 – 50%) and 5 (>50%).

HCC Hepatocellular carcinoma; PTLD Post-transplant lymphoproliferative disease; LCL Lymphoblastoid cell line
